# Hepatic Unsaturated Fatty Acids Are Linked to Lower Degree of Fibrosis in Non-alcoholic Fatty Liver Disease

**DOI:** 10.3389/fmed.2021.814951

**Published:** 2022-01-10

**Authors:** Michael Fridén, Fredrik Rosqvist, Håkan Ahlström, Heiko G. Niessen, Christian Schultheis, Paul Hockings, Johannes Hulthe, Anders Gummesson, Alkwin Wanders, Fredrik Rorsman, Ulf Risérus, Johan Vessby

**Affiliations:** ^1^Department of Public Health and Caring Sciences, Clinical Nutrition and Metabolism, Uppsala University, Uppsala, Sweden; ^2^Department of Surgical Sciences, Radiology, Uppsala University, Uppsala, Sweden; ^3^Antaros Medical AB, BioVenture Hub, Mölndal, Sweden; ^4^Department of Translational Medicine and Clinical Pharmacology, Boehringer Ingelheim Pharma GmbH & Co. KG, Biberach, Germany; ^5^MedTech West, Chalmers University of Technology, Gothenburg, Sweden; ^6^Department of Clinical Genetics and Genomics, Region Västra Götaland, Sahlgrenska University Hospital, Gothenburg, Sweden; ^7^Department of Pathology, Aalborg University Hospital, Aalborg, Denmark; ^8^Department of Medical Sciences, Gastroenterology and Hepatology, Uppsala University, Uppsala, Sweden

**Keywords:** lipids, biomarkers, fatty acids, fibrosis, NAFLD

## Abstract

**Background:** The hepatic lipidome of patients with early stages of non-alcoholic fatty liver disease (NAFLD) has been fairly well-explored. However, studies on more progressive forms of NAFLD, i.e., liver fibrosis, are limited.

**Materials and methods:** Liver fatty acids were determined in cholesteryl esters (CE), phospholipids (PL), and triacylglycerols (TAG) by gas chromatography. Cross-sectional associations between fatty acids and biopsy-proven NAFLD fibrosis (*n* = 60) were assessed using multivariable logistic regression models. Stages of fibrosis were dichotomized into none-mild (F0–1) or significant fibrosis (F2–4). Models were adjusted for body-mass index (BMI), age and patatin-like phospholipase domain-containing protein 3 (PNPLA3 rs738409) (I148M) genotype. A secondary analysis examined whether associations from the primary analysis could be confirmed in the corresponding plasma lipid fractions.

**Results:** PL behenic acid (22:0) was directly associated [OR (95% CI): 1.86 (1.00, 3.45)] whereas PL docosahexaenoic acid (22:6n-3) [OR (95% CI): 0.45 (0.23, 0.89)], TAG oleic acid (18:1n-9) [OR (95% CI): 0.52 (0.28, 0.95)] and 18:1n-9 and vaccenic acid (18:1n-7) (18:1) [OR (95% CI): 0.52 (0.28, 0.96)] were inversely associated with liver fibrosis. In plasma, TAG 18:1n-9 [OR (95% CI): 0.55 (0.31, 0.99)], TAG 18:1 [OR (95% CI): 0.54 (0.30, 0.97)] and PL 22:0 [OR (95% CI): 0.46 (0.25, 0.86)] were inversely associated with liver fibrosis.

**Conclusion:** Higher TAG 18:1n-9 levels were linked to lower fibrosis in both liver and plasma, possibly reflecting an altered fatty acid metabolism. Whether PL 22:6n-3 has a protective role, together with a potentially adverse effect of hepatic 22:0, on liver fibrosis warrants large-scale studies.

## Introduction

Non-alcoholic fatty liver disease (NAFLD) affects more than 25% of the world's population (1). Its wide clinical spectrum encompasses simple isolated steatosis, non-alcoholic steatohepatitis (NASH) with varying degree of fibrosis, cirrhosis, and hepatocellular carcinoma (HCC). NAFLD is closely associated with the incidence of type-2 diabetes mellitus, cardiovascular disease, liver-related morbidity and all-cause mortality and is currently the second leading cause of end-stage liver disease (2). Large observational studies have indicated that a higher stage of fibrosis, ranging from significant fibrosis (stage 2) to cirrhosis (stage 4), is the strongest histological predictor of liver-related and all-cause mortality in NAFLD (3, 4). It is thus important to enhance our understanding of the underlying pathophysiology of fibrotic scarring in patients with NAFLD.

Fatty acids and other lipid species have been shown to be implicated in human NAFLD development, and more specifically in the early stages of liver fat accumulation (5–10). Rosqvist et al. showed that a hypercaloric diet rich in saturated fatty acids (SFA) resulted in a marked increase in intrahepatic fat in comparison to a diet rich in polyunsaturated fatty acids (PUFA) (7, 8). The SFA-rich diet also increased circulating ceramides, lipid molecules that may trigger fibrogenesis by activating lipogenic and pro-inflammatory pathways and hepatic stellate cells (HSC) (5). However, the role for dietary fatty acids and/or endogenously synthesized fatty acids in NAFLD fibrogenesis remains unclear. Although several cross-sectional studies have investigated associations between plasma and hepatic fatty acids and the prevalence of NASH (6, 11–16), studies on liver-derived fatty acids and biopsy-proven fibrosis in patients with NAFLD are limited. Furthermore, as liver biopsies are difficult and time-consuming to obtain, prone to sampling variability and may put patients at risk of complications, it is important to find reliable and reproducible non-invasive biomarkers (e.g., circulating fatty acids) of liver fibrosis. Such biomarkers, either alone or as complements to existing scores, may add important value in diagnosing NAFLD fibrosis and for monitoring disease progression and treatment response.

The primary aim of this study was therefore to investigate associations between liver fatty acids measured in three different lipid fractions and biopsy-proven liver fibrosis in patients with NAFLD. A secondary aim was to examine whether associations between liver fatty acids and fibrosis could be confirmed in plasma-derived fatty acids.

## Materials and Methods

The current analysis is part of the AM-02 NASH study, a cross-sectional study with the main objective to evaluate the ability of non-invasive imaging biomarkers to discriminate between NASH and NAFL for the purpose to use in future clinical trials of NASH therapeutics. Subjects were recruited from the Departments of Gastroenterology and Hepatology and from the Swedish CArdioPulmonary BioImage Study “SCAPIS” (17). A total of *n* = 134 individuals were screened for eligibility. Inclusion criteria included a signed informed consent, an age between 18 and 70, clinically suspected NAFLD and at least one of the following: imaging indicative of NAFLD, alanine aminotransferase (ALT) more than 1.5 × upper limit of normal, caspase-cleaved fragment of cytokeratin 18 (CK18 M30) concentration >180 U/L and/or a biopsy showing NAFLD within 3 months prior to screening visit. Exclusion criteria included clinical or histological evidence of alcoholic liver disease, regular and excessive use of alcohol (>30 grams/day for men and >20 grams/day for women), drug addiction, history of any other liver disease, treatment with corticosteroids or immunosuppressive therapy within 10 weeks before screening visit, human immunodeficiency virus (HIV) infection, standard exclusion criteria for magnetic resonance imaging (MRI) (BMI > 40, claustrophobia, metal in body, renal insufficiency) and liver biopsy (any bleeding disorder or medication with anticoagulants) and pregnant and/or breastfeeding women. A total of *n* = 68 subjects (*n* = 15 with NAFL and *n* = 53 with NASH) were eligible for continuation in the study. From these, a sample size of *n* = 60 from those subjects who provided liver tissue for fatty acid composition analysis was included in the current analysis of the AM-02 NASH study. A complete flow-chart of the study is depicted in Supplementary Figure 1. The study was approved by the Swedish Ethical Review Authority.

### Liver and Plasma Fatty Acids (Exposure)

Hepatic and plasma fatty acids were analyzed using gas chromatography (Agilent Technologies system, 7890B), as described previously (18). A total of 14 fatty acids in both cholesteryl esters (CE) and triacylglycerols (TAG) and 19 fatty acids in phospholipids (PL) were analyzed. For each lipid fraction, five fatty acid ratios reflecting the activity of desaturase and elongase enzymes were analyzed: stearoyl-coA desaturase 1 index (SCD-1) [palmitoleic acid (16:1n-7)/palmitic acid (16:0)], Δ5 desaturase (D5D) [arachidonic acid (AA) (20:4n-6)/dihomo-γ-linolenic acid (20:3n-6)], Δ6 desaturase (D6D) [γ-linolenic acid (18:3n-6)/linoleic acid (LA) (18:2n-6)], stearic acid (18:0)/palmitic acid (16:0) and AA/LA (20:4n-6/18:2n-6). In addition, for each lipid fraction individual fatty acids were pooled into five separate fatty acid classes: total SFA, total monounsaturated fatty acids (MUFA), total PUFA, total n-3 PUFA, and total n-6 PUFA.

### Liver Fibrosis (Outcome)

The main outcome measure of this analysis was liver fibrosis. After a fast of at least 6 h, the study subjects underwent an ultrasound-guided liver biopsy. Liver biopsies were assessed by two experienced liver pathologists individually blinded to clinical, radiology and biomarker data using the steatosis, activity and fibrosis (SAF) histological scoring system (19). If the score differed between the two pathologists, the sample was re-evaluated in consensus. The stage of fibrosis was assessed as F0 (none), F1 (1a or 1b perisinusoidal zone 3 or 1c portal fibrosis), F2 (perisinusoidal and periportal fibrosis without bridging), F3 (bridging fibrosis) and F4 (cirrhosis). For the purpose of this study, stages F0 and F1 [none to mild fibrosis (F0–1)] as well as F2, F3, and F4 [significant fibrosis (F2–4)] were combined into two separate groups.

### BMI, Age, and PNPLA3 (I148M) (Covariates)

Potential confounders were chosen based on the background literature and a directed acyclic graph (DAG). The DAG was constructed in Dagitty and can be found in Supplementary Figure 2 (20). Sex, age, body mass index (BMI), patatin-like phospholipase domain-containing protein 3 (PNPLA3) rs738409 (I148M, C/G) genotype, transmembrane 6 superfamily member 2 (TM6SF2) rs58542926 (E167K, C/T) genotype and diet were identified as potential confounders. However, due to the small sample size of *n* = 60 and the risk of overfitting the logistic regression model, only the most relevant confounders were included in the final model. Included covariates were: BMI (continuous), age (continuous) and PNPLA3 rs738409 (I148M, C/G) genotype (categorical). BMI was calculated as the weight (kg) divided by the height (m) squared. Age was captured at the first screening visit, together with other demographics using a self-administered questionnaire. PNPLA3 rs738409 (I148M) was genotyped after DNA extraction from blood samples collected at visit 3 using the TaqMan^®^ PCR method, according to manufacturer's instructions. Genotypes were subsequently dichotomized into CC or CG/GG groups. Sex was excluded based on conflicting findings regarding its association with liver fibrosis (20). TM6SF2 rs58542926 (E167K, C/T) was excluded based on the lack of studies examining the association of this single nucleotide polymorphism (SNP) on hepatic fatty acid composition. Lastly, diet was excluded due to missing information on this variable.

### Liver Fat, NASH and Clinical Laboratory Measures

Liver fat was assessed by MRI (Achieva, Philips Healthcare, Best, Netherlands) after 6 h of fasting, as described previously (21). Histological grading of steatosis, lobular inflammation and hepatocyte ballooning was assessed based on the SAF histological scoring system (19). Stages of steatosis were classified as 0 (<5% of hepatocytes with large or medium-sized lipid droplets), 1 (5–33% of hepatocytes with large or medium-sized lipid droplets), 2 (34–66% of hepatocytes with large or medium-sized lipid droplets) and 3 (>67% of hepatocytes with large or medium-sized lipid droplets). Ballooning was categorized as 0 (normal hepatocytes with cuboidal shape and pink eosinophilic cytoplasm), 1 (presence of clusters of hepatocytes with a rounded shape and pale cytoplasm usually reticulated) and 2 (same as 1 with some enlarged hepatocytes, at least 2-fold that of normal cells). Lobular inflammation was defined as a focus of two or more inflammatory cells within the lobule, where foci were counted at 20x magnification (0: none; 1: ≤ 2 foci per 20x; 2: >2 foci per 20x). NASH was defined when at least one point was given to both lobular inflammation and ballooning in the presence of steatosis (at least one point). Clinical variables from fasting blood samples collected at the same clinic visit as the liver biopsy (Supplementary Figure 1), were assessed by standard laboratory techniques at Uppsala University Hospital.

### Statistical Analysis

The primary analysis of this study was to examine associations between liver-derived fatty acids and NAFLD fibrosis. A secondary analysis was to examine whether potential associations between liver-derived fatty acids and fibrosis could be confirmed in plasma fatty acids. Both of these analyses were decided upon *a priori*. Further *post-hoc* analyses included (1) pooling individual liver and plasma fatty acids into their respective fatty acid classes and examine their associations with liver fibrosis (2) performing principal component analyses (PCA) for both liver and plasma fatty acids and model the first principal component (PC1) for each lipid fraction with liver fibrosis in multivariable logistic regression analyses. Normal distribution was assessed using the Shapiro-Wilk W test and when appropriate, skewed distributed continuous variables (W <0.95) were logarithmically transformed before analysis or analyzed non-parametrically. Two-sample *t*-tests or non-parametric Mann-Whitney U tests were performed for between-group comparisons (F0–1 vs. F2–4) in population characteristics and liver fatty acid composition. Homogeneity of variance was assessed through visual inspection of the standard deviations or by box-plots. Spearman rank correlations were performed between liver- and plasma-derived fatty acids.

Multivariable logistic regression analyses, adjusted for age, BMI and PNPLA3 (I148M) genotype, were performed for each liver-derived fatty acid, fatty acid ratio and pooled fatty acids in the three lipid fractions (CE, PL, TAG) and the prevalence of significant fibrosis (F2–4) (dichotomized outcome). BMI, age and all fatty acids were treated as continuous variables whereas the PNPLA3 (I148M) genotype was treated as a categorical nominal variable (CC vs. CG/GG). Odds ratios with 95% confidence intervals were calculated from the multivariable logistic regression models for each standard deviation change in fatty acid proportion. Further *post-hoc* sensitivity analyses including both sex (categorical) and TM6SF2 (E167K) (dichotomized into CC or CT/TT) in addition to the *a priori* determined confounders; BMI, age and PNPLA3 (I148M) were performed for those hepatic fatty acids that were statistically significantly associated with liver fibrosis in the primary analysis and for all plasma fatty acids in the secondary analysis. Multicollinearity was inspected using correlation matrices between covariates in the logistic regression model and a correlation coefficient of 0.8 or more was predetermined to be indicative of multicollinearity. None of the covariates in the model had any missing data, hence imputation was not needed.

No correction for multiple hypothesis testing was applied due to the exploratory nature of this study. All statistical analyses were performed using JMP software version 15.1.0 (SAS Institute, Inc) and a *P* < 0.05 was set as the significance level.

## Results

Population characteristics are shown in Table 1. The mean ages of F0–1 (*n* = 36) and F2–4 (*n* = 24) were 58.5 and 57.0 years, respectively. The proportions of males and females were similar between the groups (58% males in F0–1 and 63% males in F2–4). There were no statistically significant differences in any of the clinical (i.e., platelets and albumin) or histological variables (i.e., liver fat, NASH prevalence, SAF ballooning and lobular inflammation) related to NAFLD severity between F0–1 subjects and F2–4 subjects, except for the degree of liver fibrosis. Mean BMI was 30.3 kg/m^2^ and 30.8 kg/m^2^ for F0–1 and F2–4, respectively. Prevalence of type-2 diabetes, hypertension and hyperlipidaemia was 43, 57, and 27% for F0–1 and 45, 59, and 18% for F2–4. The distribution of the PNPLA3 genotype [CC/(CC/CG)] was 44/56% for F0–1 and 46/54% for F2–4.

**Table 1:** Population characteristics^a^.

	**F0–1 (*n* = 36)**	**F2–4 (*n* = 24)**	***P*-value**
Age (years)	58.5 (53.5–63.0)	57.0 (42.8–66.5)	0.95^*^
Sex [*n* (%) male/female]	21 (58)/15 (42)	15 (63)/9 (38)	0.75
BMI (kg/m^2^)	30.3 ± 3.8	30.8 ± 3.0	0.57
Type-2 diabetes [*n* (%)]^**^	13 (43)	10 (45)	0.88
Hypertension [*n* (%)]^**^	17 (57)	13 (59)	0.86
Hyperlipidaemia [*n* (%)]^**^	8 (27)	4 (18)	0.47
Platelets (10^9^/L)	232.6 ± 53.5	235.0 ± 61.6	0.87
Albumin (g/L)	39.1 ± 2.9	40.3 ± 2.9	0.12
Liver fat (%)	14.7 (8.8–24.1)	17.9 (11.2–20.4)	0.32
SAF steatosis [*n* (%) 1/2/3]	14 (39)/11 (31)/11 (31)	6 (25)/11 (46)/7 (29)	0.41
PNPLA3 (I148M) [*n* (%) CC/(CG/GG)]	16 (44)/20 (56)	11 (46)/13 (54)	0.92
NASH [*n* (%)]	25 (69)	21 (88)	0.11
SAF ballooning [*n* (%) 0/1/2]	3 (8)/30 (83)/3 (8)	0/20 (83)/4 (17)	0.32
SAF lobular inflammation [*n* (%) 0/1/2]	10 (28)/26 (72)/0	3 (13)/20 (83)/1 (4)	0.16
Fibrosis stage [*n* (%) 0/1/2/3/4]	4 (11)/32 (89)/0/0/0	0/0/19 (79)/3 (13)/2 (8)	

a *Data are presented as mean ± SD, % or as median (IQR) for skewed distributed variables*.

*BMI, Body mass index; F0–1, Fibrosis stages 0–1; F2–4, Fibrosis stages 2–4; NASH, Non-alcoholic steatohepatitis; PNPLA3, Patatin-like phospholipase domain-containing protein 3; SAF, steatosis, activity and fibrosis*.

*SAF ballooning categories 0–1 and SAF lobular inflammation categories 1–2 were combined into one category to satisfy the assumptions of the Chi-2 test.*

* *Analyzed using a non-parametric Mann-Whitney U test*.

** *n = 52*.

### Liver Fatty Acid Proportions Between F0–1 and F2–4

**Figure 1:**
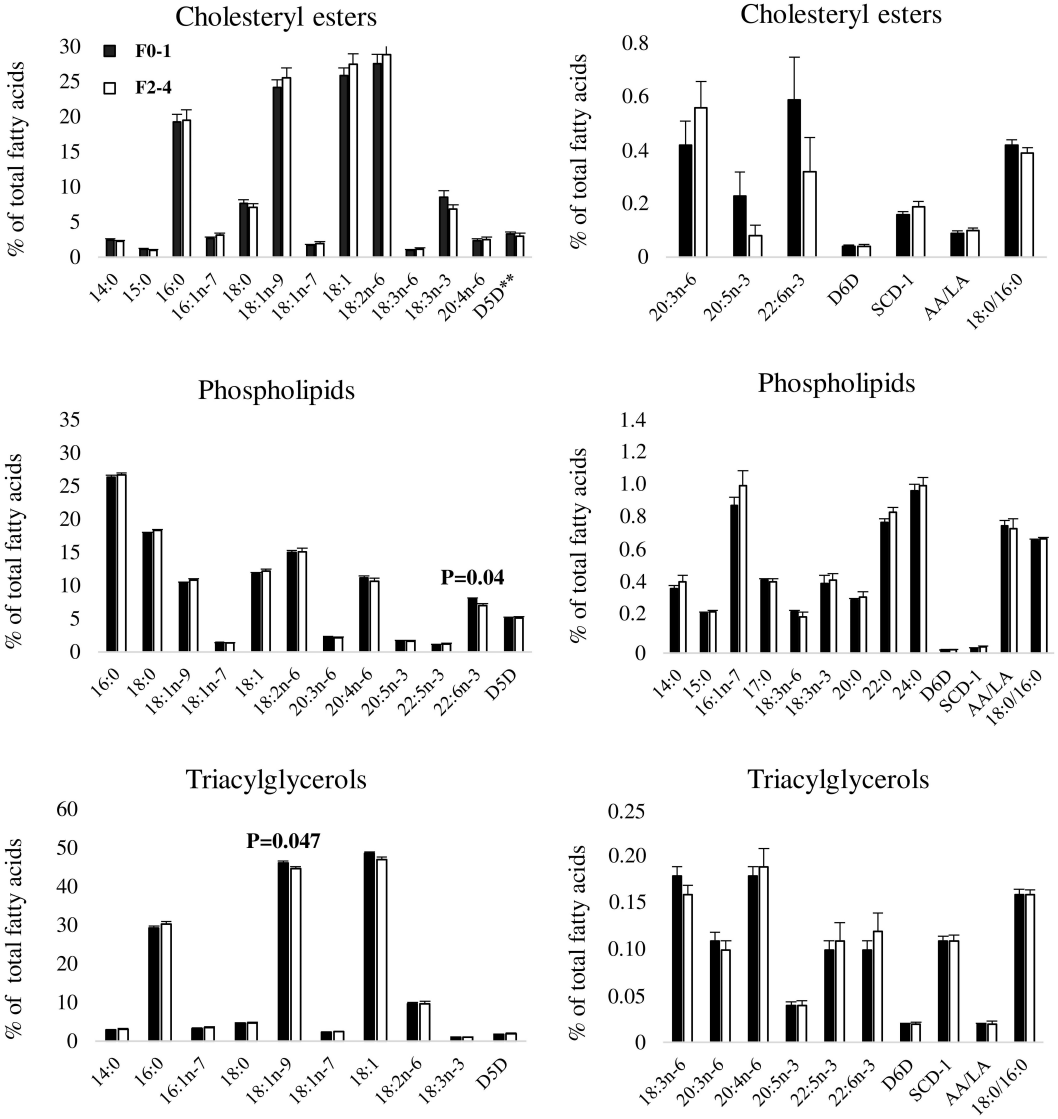
Proportions of liver fatty acids between F0–1 and F2–4. Data are expressed as means ± SEM. Black bars represent fibrosis stages 0–1 (F0–1) and white bars represent fibrosis stages (F2–4), as indicated by the legend in the top left corner of the first bar graph. Due to large differences in proportions of fatty acids, each lipid fraction is divided into two separate graphs to enhance visibility. 14:0, myristic acid; 15:0, pentadecaenoic acid; 16:0, palmitic acid; 16:1n-7, palmitoleic acid; 17:0, heptadecaenoic acid; 18:0, stearic acid; 18:1n-9, oleic acid; 18:1n-7, vaccenic acid; 18:1, oleic acid combined with vaccenic acid; 18:2n-6, linoleic acid; 18:3n-6, γ-linolenic acid; 18:3n-3, α-linolenic acid; 20:0, arachidic acid; 20:3n-6, dihomo-γ-linolenic acid; 20:4n-6, arachidonic acid; 20:5n-3, eicosapentaenoic acid; 22:0, behenic acid; 22:5n-3, docosapentaenoic acid; 22:6n-3, docosahexaenoic acid; 24:0, lignoceric acid; SCD-1, stearoyl-coA desaturase; D5D, delta 5 desaturase; D6D, delta 6 desaturase; AA/LA, arachidonic acid/linoleic acid. SCD-1, D5D and D6D are estimated using fatty acid product-to-precursor ratios: 16:1n-7/16:0 (SCD-1), 20:4n-6/20:3n-6 (D5D), 18:3n-6/18:2n-6 (D6D). **n(F0–1) = 19, n(F2–4) = 16 due to 25 zero-values of 20:3n-6.

Liver fatty acids and fatty acid ratios were analyzed in three lipid fractions (CE, PL, TAG) and were subsequently compared between subjects with F0–1 and F2–4. There were no statistically significant differences in proportions of liver CE fatty acids between F0–1 and F2–4. However, proportional differences were observed for PL docosahexaenoic acid (22:6n-3) (F0–1: 7.92 (SEM 0.25), F2–4: 6.97 (SEM 0.37), *P* = 0.04) and TAG oleic acid (18:1n-9) [F0–1: 46.13 (SEM 0.43), F2–4: 44.61 (SEM 0.61), *P* = 0.047] (Figure 1).

### Associations Between Liver Fatty Acids and Liver Fibrosis

**Figure 2:**
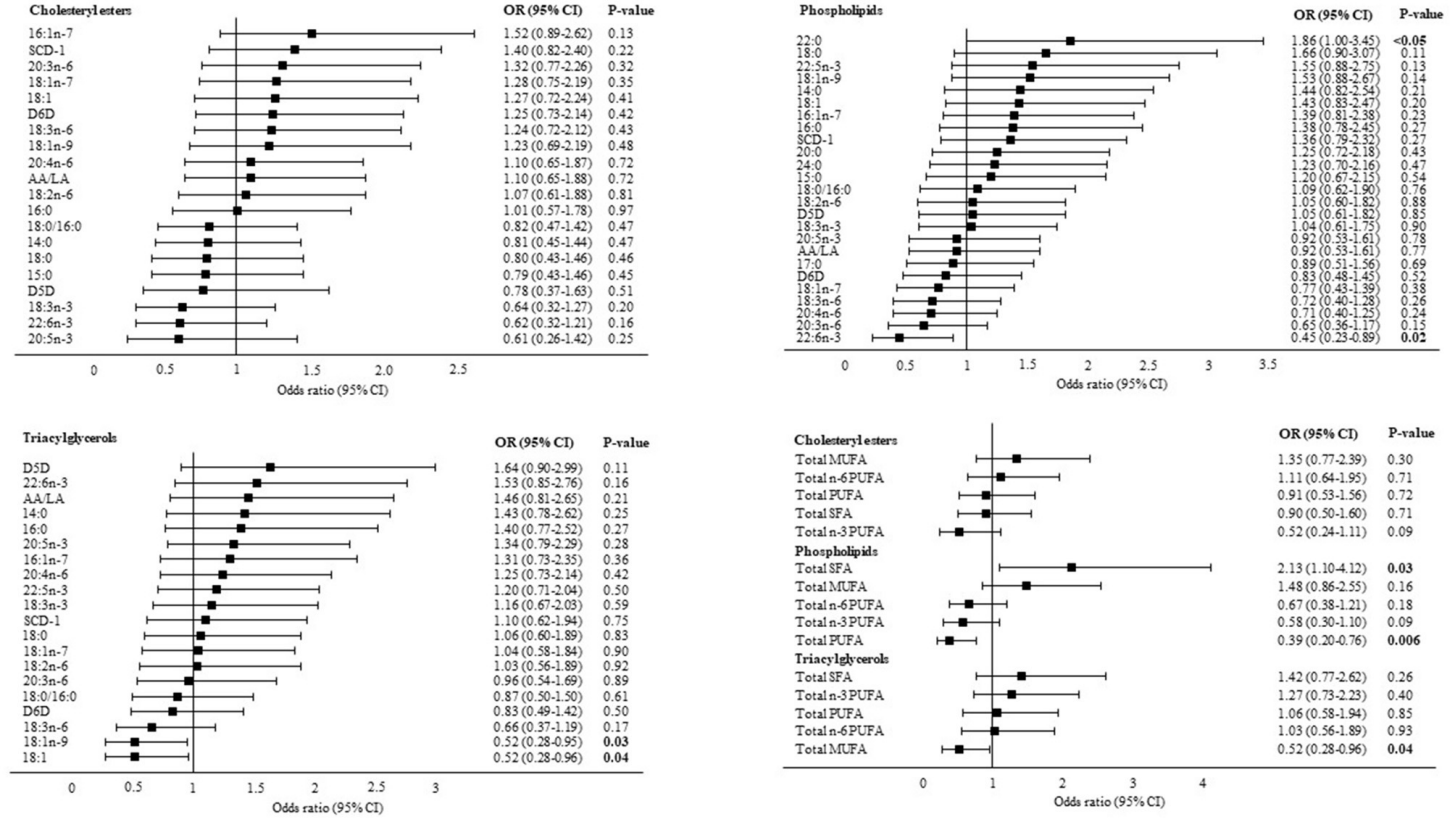
Associations between liver fatty acids and significant liver fibrosis. Data are presented as adjusted odds ratios **(OR)** with 95% confidence intervals **(CI)** and *P*-values for each standard deviation change in liver fatty acid proportions. OR are adjusted for BMI, age and PNPLA3 (I148M) genotype. 14:0, myristic acid; 15:0, pentadecaenoic acid; 16:0, palmitic acid; 16:1n-7, palmitoleic acid; 17:0, heptadecaenoic acid; 18:0, stearic acid; 18:1n-9, oleic acid; 18:1n-7, vaccenic acid; 18:1, oleic acid combined with vaccenic acid; 18:2n-6, linoleic acid; 18:3n-6, γ-linolenic acid; 18:3n-3, α-linolenic acid; 20:0, arachidic acid; 20:3n-6, dihomo-γ-linolenic acid; 20:4n-6, arachidonic acid; 20:5n-3, eicosapentaenoic acid; 22:0, behenic acid; 22:5n-3, docosapentaenoic acid; 22:6n-3, docosahexaenoic acid; 24:0, lignoceric acid; SCD-1, stearoyl-coA desaturase; D5D, delta 5 desaturase; D6D, delta 6 desaturase; AA/LA, arachidonic acid/linoleic acid. SCD-1, D5D and D6D are estimated using fatty acid product-to-precursor ratios: 16:1n-7/16:0 (SCD-1), 20:4n-6/20:3n-6 (D5D), 18:3n-6/18:2n-6 (D6D). D5D in cholesteryl esters: n(F0–1) = 19, n(F2–4) = 16 due to 25 zero-values of 20:3n-6.

In multivariable logistic regression analyses adjusted for BMI, age and PNPLA3 (I148M) genotype, PL behenic acid (22:0) (OR: 1.86, 95% CI: 1.0, 3.45, *P* < 0.05) was directly associated with liver fibrosis whereas PL 22:6n-3 (OR: 0.45, 95% CI: 0.23, 0.89, *P* = 0.02), TAG 18:1n-9 (OR: 0.52, 95% CI: 0.28, 0.95, *P* = 0.03) and TAG 18:1n-9 combined with vaccenic acid (18:1n-7) (18:1) were inversely associated with liver fibrosis. Pooling individual fatty acids into their respective fatty acid classes demonstrated a positive association between total PL SFA and liver fibrosis (OR: 2.13, 95% CI: 1.10, 4.12, *P* = 0.03) and inverse associations between total PL PUFA (OR: 0.39, 95% CI: 0.20, 0.76, *P* = 0.006), total TAG MUFA (OR: 0.52, 95% CI: 0.28, 0.96, *P* = 0.04) and liver fibrosis (Figure 2).

### Associations Between Plasma Fatty Acids and Liver Fibrosis

**Figure 3:**
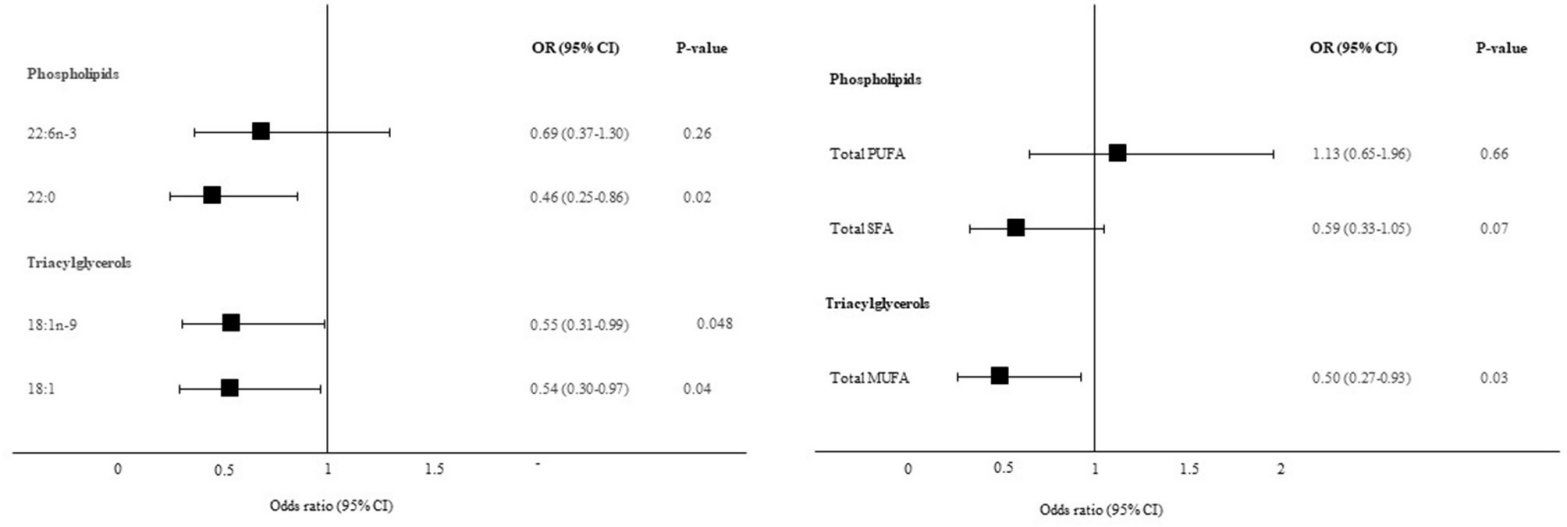
Association between plasma fatty acids and significant liver fibrosis. Data are presented as adjusted odds ratios **(OR)** with 95% confidence intervals **(CI)** and *P*-values for each standard deviation change in plasma fatty acid proportions. OR are adjusted for BMI, age and PNPLA3 (I148M) genotype. 18:1n-9, oleic acid; 18:1, oleic acid combined with vaccenic acid; 22:0, behenic acid; 22:6n-3, docosahexaenoic acid.

The corresponding plasma fatty acids to those liver-derived fatty acids that were associated with liver fibrosis in Figure 2, were further included in multivariable logistic regression models, adjusted for BMI, age and PNPLA3 (I148M) genotype. Both TAG 18:1n-9 (OR: 0.55, 95% CI: 0.31, 0.99, *P* = 0.048) and TAG 18:1 (OR: 0.54, 95% CI: 0.30, 0.97, *P* = 0.04) demonstrated similar associations with liver fibrosis in plasma as for in the liver. Plasma TAG 18:1n-9 correlated strongly with liver TAG 18:1n-9 (Spearman rho = 0.73, *P* < 0.0001). Interestingly, PL 22:0 showed the opposite relationship with liver fibrosis in plasma as for in the liver (OR: 0.46, 95% CI: 0.25, 0.86, *P* = 0.02). Plasma PL 22:0 was not correlated with liver PL 22:0 (Spearman rho = 0.07, *P* = 0.58). No association was observed for PL 22:6n-3. Total plasma TAG MUFA was inversely associated with liver fibrosis in *post-hoc* analyses (OR: 0.50, 95% CI: 0.27, 0.93, *P* = 0.03). No associations were observed for plasma PL PUFA or plasma PL SFA and liver fibrosis (Figure 3).

### Principal Components of Fatty Acids in Plasma and Liver

Although a trend was observed between PC1 in plasma PL and liver fibrosis in multivariable logistic regression analyses (adjusted for BMI, age and PNPLA3 (I148M) genotype) (OR: 1.18, 95% CI: 0.95, 1.45, *P* = 0.13), no further associations were observed (data not shown). PC1 was characterized by lower proportions of pentadecanoic acid (15:0), heptadecanoic acid (17:0) and very long-chain SFA and higher proportions of 16:0, 16:1n-7, 18:1n-9, 18:1, 18:3n-6, D5D and SCD-1.

### *Post-hoc* Sensitivity Analyses

None of the associations between liver or plasma fatty acids and liver fibrosis were markedly attenuated when including sex and TM6SF2 (E167K) as additional confounders in the logistic regression models (Supplementary Table 1).

## Discussion

In this cross-sectional study of subjects with biopsy-proven NAFLD, we observed several links between the composition of liver fatty acids and fibrosis. A positive association between liver PL 22:0 and inverse associations between liver PL 22:6n-3, TAG 18:1n-9 and TAG 18:1 and liver fibrosis were observed. These associations were confirmed in plasma TAG 18:1n-9 and 18:1, however an inverse association was observed for plasma PL 22:0. Furthermore, *post-hoc* analyses demonstrated a positive association between liver PL SFA and inverse associations between liver PL PUFA and liver TAG MUFA and liver fibrosis. The latter findings were confirmed in plasma TAG MUFA but not in plasma PL PUFA or PL SFA.

Our finding of a PUFA/MUFA-depleted, SFA-enriched liver in subjects with NAFLD fibrosis, characterized by lower proportions of 22:6n-3 and 18:1n-9 and higher proportions of 22:0 is to some extent in accordance with previous cross-sectional studies on hepatic lipid profiles in NAFLD. A relatively consistent pattern from these studies is that subjects with NAFL/NASH are characterized by PUFA-depleted livers, with lower proportions of 20:4n-6, eicosapentaenoic acid (20:5n-3) and 22:6n-3 (13, 15, 16). Notably, Kotronen et al. demonstrated an inverse correlation between hepatic PUFA (both 22:6n-3 and 18:2n-6) and free fatty acid 16:0 in severely obese patients, suggesting that PUFA might be implicated in the pathogenesis of steatosis (22). However, when comparing liver fatty acid profiles of subjects with NASH vs. NAFL, these differences are largely diminished, possibly indicating a greater role for fatty acids in the early stages of NAFLD (6, 12, 15, 16).

The relationship between fatty acids and NAFLD severity is complex. In the present study, we identified liver-derived fatty acids that were independently associated with liver fibrosis. Among these were 22:6n-3, an omega-3 PUFA that has been reported to be depleted in livers of patients with alcoholic cirrhosis (23), and which potentially could inhibit fibrogenesis through multiple mechanisms. These include inhibition of lipogenic pathways and the production of pro-inflammatory eicosanoids as well as suppression of HSC activation (24, 25). Due to the limited conversion rate from α-linolenic acid (18:3n-3) and 20:5n-3, plasma and tissue levels of 22:6n-3 mainly reflect dietary intake of marine sources, such as fatty fish and fish-oil supplements (26). On the contrary however, human supplementation trials of omega-3 fatty acids in NAFLD fibrosis have been few and findings have been mixed, with durations ranging from 6 to 12 months and with combined doses of both 22:6n-3 and 20:5n-3 ranging from 600 to 3,600 mg/day (27, 28). This apparent heterogeneity makes it difficult to draw any firm conclusions regarding the role of 22:6n-3 supplementation in NAFLD fibrosis. Longer-term follow-up studies with higher doses of isolated 22:6n-3 are warranted (29). Furthermore, as 22:6n-3 is an established biomarker of fatty fish intake, attention should be directed to investigate the potential role for diet in the treatment and prevention of NAFLD fibrosis. Lastly, altered desaturation and elongation of 22:6n-3 in liver fibrosis cannot be ruled out.

Interestingly, we also found opposite directions of associations between liver PL and plasma PL 22:0 and liver fibrosis. 22:0 is a very long-chain SFA that has been inversely associated with the incidence of type-2 diabetes in studies using circulating fatty acids (30), supporting our findings of an inverse association between plasma 22:0 and liver fibrosis. However, no studies have yet assessed these very long-chain SFA in human liver tissue in relation to cardiometabolic diseases. Jang et al. recently showed, using an arteriovenous technique combined with a metabolomics approach in pigs, that 22:0 constituted a significant part of metabolites produced from lung tissue, indicating that circulating very long chain SFA in humans may partially reflect extrahepatic tissue metabolism (31). This finding is further supported by the lack of correlation between liver and plasma PL 22:0 in our study (Spearman rho = 0.07, *P* = 0.58). The relation between very long chain SFA and NAFLD warrants further attention in future studies.

Notably, 18:1n-9 and the combined 18 carbon MUFA 18:1 in both liver and plasma TAG were inversely associated with liver fibrosis. Although circulating 18:1n-9 might partially reflect dietary intake of MUFA-rich sources (e.g., olive oil or rapeseed oil) (32, 33), 18:1n-9 in plasma is primarily endogenously synthesized from SFA by SCD-1 and has been associated with the incidence of type-2 diabetes in a pooled sample of 17 prospective studies and with elevated liver enzymes in one cross-sectional study (34, 35). However, the vast majority of these studies have assessed 18:1n-9 in either PL or CE and not in TAG. In the Finnish METSIM cohort however, 18:1n-9 was measured in circulating TAG, demonstrating a non-significant inverse association with the incidence of type-2 diabetes (37). These findings are indirectly supported by the non-significant positive associations between 18:1n-9 and liver fibrosis in CE and PL in our study. This highlights the importance of cautious interpretation when extrapolating fatty acids from one lipid fraction to another. As further support, Araya et al. observed an increase of 18:1n-9 in total lipids, but not in TAG, in patients with NAFL and NASH vs. controls (15). Importantly, TAG 18:1n-9 in the liver correlated strongly with TAG 18:1n-9 in plasma in our study (Spearman rho = 0.73, *P* < 0.0001). The inverse association between TAG 18:1n-9 and liver fibrosis could be considered contradictory, however, it might reflect an enhanced desaturation and elimination of lipotoxic 16:0 through enhanced SCD-1 activity (36). Taken together, our findings suggest that plasma fatty acids could potentially be used as biomarkers for discriminating patients with NAFLD fibrosis and encourage further large-scale studies in the area.

There are several limitations worthy of consideration. First, the cross-sectional design makes it impossible to infer causality from our findings. Secondly, since the AM-02 NASH study was not designed to primarily address research questions posed in this study, lack of associations between fatty acids and liver fibrosis might be explained by small sample sizes and hence lower statistical power, indicated by the wide confidence intervals of the estimates. Lastly, missing information on diet might have contributed to residual confounding and thereby distorted the associations between fatty acids and liver fibrosis. At the same time, there are several strengths worth highlighting. Firstly, liver fibrosis was diagnosed from liver biopsies by two independent liver pathologists, and although biopsies are prone to sampling errors, histological grading remains the gold standard in assessing liver fibrosis in NAFLD. Secondly, fatty acids were measured in three different lipid fractions in both liver tissue and plasma, thereby allowing us to examine associations over multiple fatty acid compartments frequently used in epidemiological studies. Lastly, the homogenous fibrosis groups, as indicated in Table 1 (clinical characteristics, biochemistry and histological scores except for fibrosis) might have contributed to reducing the risk of residual confounding. Importantly, due to the small sample size and multiple hypotheses tests, our findings should be interpreted as exploratory and hypothesis generating.

In conclusion, TAG 18:1n-9 and the combined TAG MUFA 18:1 demonstrated inverse associations with significant liver fibrosis in both liver and plasma, whereas PL 22:0 showed the opposite relationships in these compartments. Large-scale studies are warranted to further investigate the role for these fatty acids as potential diagnostic biomarkers of NAFLD fibrosis. In addition, PL 22:6n-3, a biomarker of fatty fish intake, was inversely associated with fibrosis in the liver. Whether dietary modifications using marine sources of 22:6n-3 may have therapeutic implications in NAFLD fibrosis prevention needs to be investigated in longitudinal studies.

## Data Availability Statement

The datasets presented in this study can be found in online repositories. The names of the repository/repositories and accession number(s) can be found at: https://osf.io/89b3n/, https://osf.io/89b3n/.

## Ethics Statement

The studies involving human participants were reviewed and approved by Swedish Ethical Review Authority. The patients/participants provided their written informed consent to participate in this study.

## Author Contributions

MF, UR, FRos, FRor, and JV designed the research. FRor, JV, HA, HN, CS, PH, JH, AG, AW, and MF conducted the research. FRor, JV, HA, HN, CS, PH, and JH provided databases. MF analyzed data. MF, FRos, UR, FRor, and JV wrote the manuscript. UR had primary responsibility for final content. All authors reviewed, revised, and approved the final manuscript.

## Funding

MF and UR were supported by the Swedish Diabetes Foundation, Swedish Heart-Lung Foundation, Swedish Research Council Formas and EXODIAB (Excellence of Diabetes Research in Sweden). FRos was supported by EXODIAB and P.O. Zetterlings foundation. HA was supported by the Swedish Research Council (2016-01040 and 2019-04756), the Swedish Heart-Lung Foundation (20170492 and 20200500) and EXODIAB. This study was supported by Antaros Medical, Boehringer Ingelheim, Swedish Research Council (2016-01040), and the Swedish Heart-Lung Foundation (20170492).

## Conflict of Interest

HN and CS are employees of Boehringer Ingelheim Pharma GmbH & Co. KG. The remaining authors declare that the research was conducted in the absence of any commercial or financial relationships that could be construed as a potential conflict of interest.

## Publisher's Note

All claims expressed in this article are solely those of the authors and do not necessarily represent those of their affiliated organizations, or those of the publisher, the editors and the reviewers. Any product that may be evaluated in this article, or claim that may be made by its manufacturer, is not guaranteed or endorsed by the publisher.
