# Supplementary material for: Hepatic Unsaturated Fatty Acids Are Linked to Lower Degree of Fibrosis in Non-alcoholic Fatty Liver Disease
Source: Front Med (Lausanne). 2022 Jan 10;8:814951. doi: 10.3389/fmed.2021.814951 (PMC8784562; doi:10.3389/fmed.2021.814951)
Supplement: Supplementary file 2 [file Data_Sheet_1.PDF]

Supplementary Material

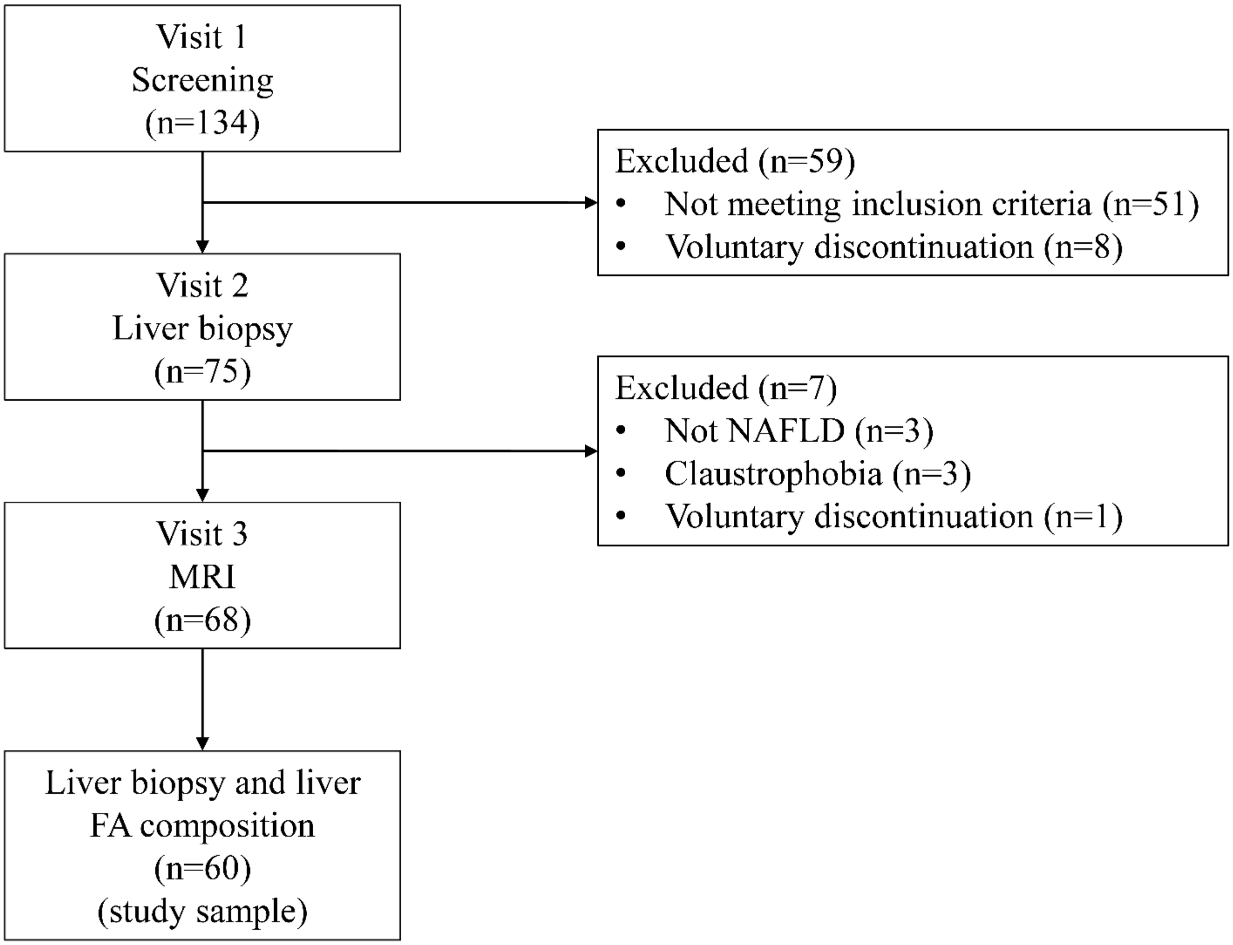

Supplementary Figure 1. Study flow chart of the AM-02 NASH study.
FA, Fatty acid; MRI, Magnetic resonance imaging; NAFLD, Non-alcoholic fatty liver disease.

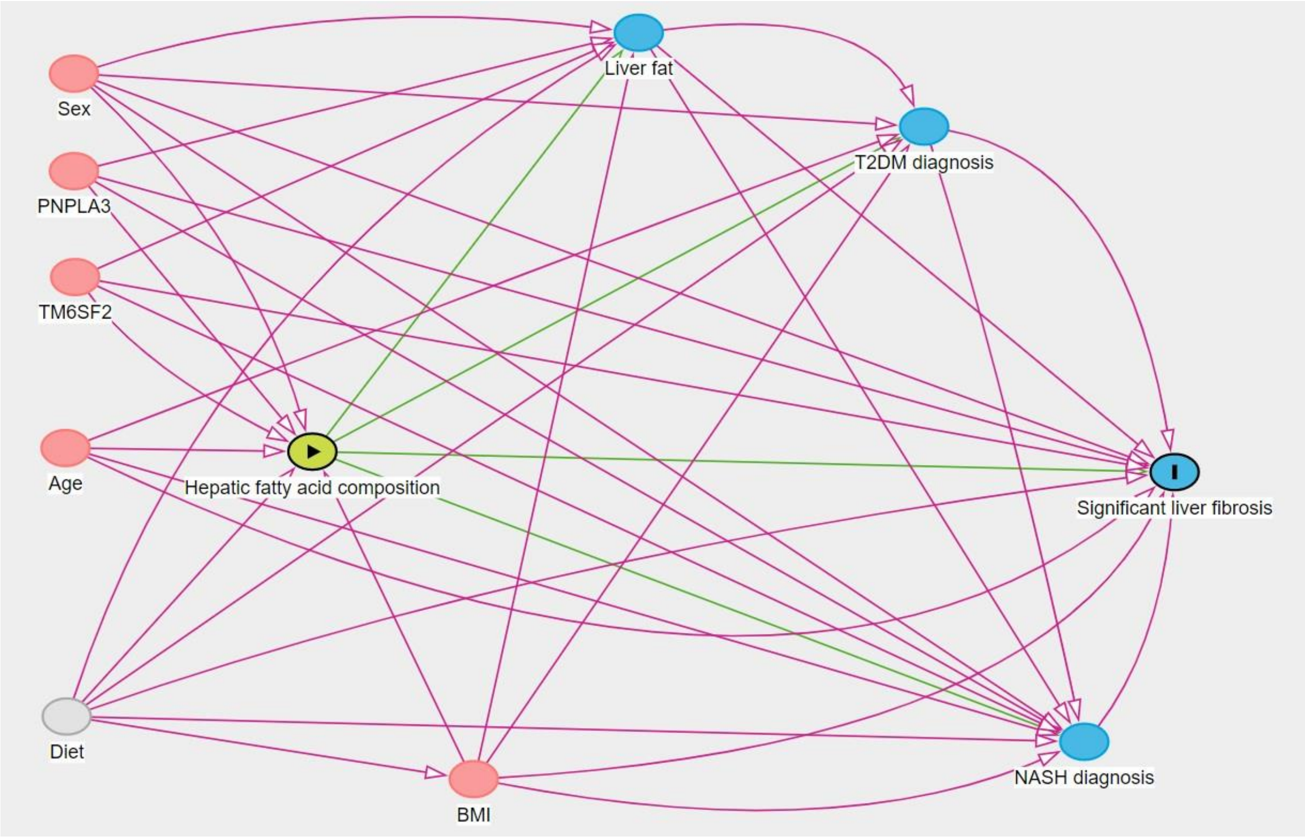

Supplementary Figure 2. A directed acyclic graph (DAG) to illustrate the causal framework for this study. The DAG was constructed in Dagitty using Dagitty.net. Potential confounders that were identified using this approach were: sex, patatin-like phospholipase domain-containing protein 3 (PNPLA3), transmembrane 6 superfamily member 2 (TM6SF2), age, body mass index (BMI) and diet. However, due to the risk of model overfitting in logistic regression analyses when working with smaller sample sizes, only the most important confounders were included in the fully adjusted model: BMI, age and PNPLA3. Diet is indicated as an unobserved variable.

Supplementary Table 1. Sensitivity analyses using multivariable logistic regression to investigate associations between hepatic and plasma fatty acids and liver fibrosis (F2-4 vs F0-1), adjusted for sex and TM6SF2 (E167K)-genotype[1] in addition to BMI, age and PNPLA3 (I148M)-genotype

Phospholipids	OR (95% CI)	P-value
22:6n-3 (liver)	0.44 (0.22-0.88)	**0.02**
22:0 (liver)	1.87 (1.00-3.47)	**<0.05**
Total SFA (liver)	2.29 (1.14-4.60)	**0.02**
Total PUFA (liver)	0.38 (0.19-0.76)	**0.007**
22:6n-3 (plasma)	0.68 (0.36-1.29)	0.24
22:0 (plasma)	0.43 (0.21-0.88)	**0.01**
Total SFA (plasma)	0.60 (0.33-1.11)	0.11
Total PUFA (plasma)	1.18 (0.67-2.08)	0.56
**Triacylglycerols**	**OR (95% CI)**	**P-value**
18:1n-9 (liver)	0.52 (0.28-0.96)	**0.04**
18:1 (liver)	0.52 (0.28-0.97)	**0.03**
Total MUFA (liver)	0.51 (0.27-0.96)	**0.04**
18:1n-9 (plasma)	0.52 (0.28-0.97)	**0.04**
18:1 (plasma)	0.50 (0.27-0.94)	**0.03**
Total MUFA (plasma)	0.45 (0.23-0.87)	**0.01**

[1]Data are presented as adjusted odds ratios (OR) with 95% confidence intervals (CI) for each standard deviation change in hepatic or plasma fatty acid proportion.
Sensitivity analyses are performed for those hepatic fatty acids that were statistically significantly associated with liver fibrosis in the primary analysis and for all plasma fatty acids in the secondary analysis. 18:1n-9, oleic acid; 18:1, oleic acid + vaccenic acid; 22:0, behenic acid; 22:6n-3, docosahexaenoic acid; MUFA, monounsaturated fatty acids; PUFA, polyunsaturated fatty acids; SFA, saturated fatty acids.
